# Effects of Hypothyroidism on the Extracellular Matrix of the Vocal Folds of Adult Female Rats

**DOI:** 10.1002/ohn.70020

**Published:** 2025-09-08

**Authors:** Welber C. Mororó, Karine V. G. de Oliveira, Jônatas B. do Amaral, Gisele Giannocco, João R. M. Martins, Noemi G. De Biase

**Affiliations:** ^1^ Department of Otorhinolaryngology, Head and Neck Surgery Universidade Federal de São Paulo/Escola Paulista de Medicina (UNIFESP/EPM) Sao Paulo Sao Paulo Brazil; ^2^ Laboratory of Molecular and Translational Endocrinology, Division of Endocrinology, Department of Medicine Universidade Federal de São Paulo/Escola Paulista de Medicina (UNIFESP/EPM) Sao Paulo Sao Paulo Brazil; ^3^ Department of Theories and Methods in Speech Therapy and Physiotherapy Pontifícia Universidade de São Paulo (PUCSP) Sao Paulo Sao Paulo Brazil

**Keywords:** collagen, elastin, hyaluronic acid, hypothyroidism, vocal folds

## Abstract

**Objective:**

The aim of the present study was to investigate the changes in the extracellular matrix of the vocal folds of female rats after induction of hypothyroidism.

**Study Design:**

Prospective and experimental study.

**Setting:**

Single tertiary center.

**Methods:**

Female rats were divided into 2 groups: hypothyroid (after thyroidectomy and treatment with methimazole) and euthyroid (control). After 8‐week experiment, the animals were euthanized and their vocal folds were removed. These were analyzed for the presence of collagen type I, collagen type III, and elastin using the enzyme‐linked immunosorbent assay technique. An hyaluronan‐binding protein isolated from bovine nasal cartilage was used to identify and isolate the hyaluronic acid for samples.

**Results:**

The hypothyroid group presented significantly increased serum TSH concentrations (*P* = .0425) and significantly decreased serum T4 concentrations (*P* = .0024) compared to the control group. The concentration of type I collagen in the vocal folds of animals in the hypothyroid group was higher than in the control group (*P* = .0432), and an increase in the concentration of type III collagen was also observed (*P* = .0339). There was no statistically significant difference in the concentrations of elastin and hyaluronic acid between the groups.

**Conclusion:**

Hypothyroidism was able to cause changes in the extracellular matrix of the vocal folds of female rats. We found an increase in the concentration of type I collagen and type III collagen in the animals with hypothyroidism. There was no difference in the concentration of elastin and hyaluronic acid between the hypothyroid and control groups.

Histologically, the human vocal fold is divided into layers: the epithelium, the lamina propria (LP) and the vocal muscle (thyroarytenoid). The epithelium is nonkeratinized and stratified. The LP is divided into a superficial layer, an intermediate layer, and a deep layer. The intermediate and deep layers are referred to as the vocal ligament, while the superficial layer of the LP is known as the Reinke space. In terms of its phonatory function, the vocal fold is divided into a body and a cover. The cover is more external and consists of the epithelium and the superficial layer of the LP. The body consists of the thyroarytenoid muscle and has a lower ability to vibrate. The vocal ligament is a transitional area between the body and the cover.^1^, ^2^

The vocal folds are composed of 2 main tissue components: the extracellular matrix and the cellular component. The extracellular matrix consists of 2 categories of macromolecules: fibrotic proteins (collagen and elastin) and material from the interstitium (proteoglycans and structural glycoproteins).^3^ Hirano^4^ described that the superficial layer of the LP consists of loose tissue with few collagen and elastic fibers. The intermediate layer of the LP consists of an increased number of elastic fibers and the deep layer of the LP consists of an increased number of collagen fibers.

Hypothyroidism is a pathological condition in which there is a reduction in thyroid hormone levels, which has a negative effect on health and can even lead to death if inadequately treated. Clinical hypothyroidism is characterized when the thyroid‐stimulating hormone (TSH) concentration is above the previously established reference values and the thyroxine (T4) concentration is below the reference values.^5^ If left untreated, it can lead to dysfunction in various tissues, which can manifest itself in different clinical presentations and cause changes in the extracellular matrix of various organs and tissues.^6^, ^7^, ^8^, ^9^

The relationship between voice symptoms and the hormonal changes in hypothyroidism has been shown in previous studies, focusing on the reduction of the fundamental frequency in hypothyroid patients.^10^, ^11^, ^12^ However, although previous studies indicate the presence of voice changes in hypothyroidism, there is no consistent data in the literature on the frequency of their occurrence. The identification of thyroid hormone receptors in the LP of the human vocal folds supports the existence of a role for these hormones in the larynx.^13^ This finding suggests that hypothyroidism can lead to changes in the extracellular matrix of the vocal folds, as has been observed in other tissues.

Given the probable influence of thyroid hormones on the nature of the extracellular matrix of the vocal folds, it is likely that this is responsible for the changes in the voice in hypothyroidism. The characterization the changes in the extracellular matrix of the vocal folds in hypothyroidism will allow a better understanding of the pathophysiology of dysphonia that occurs in these cases. We hypothesized that hypothyroidism can lead to changes in the basic structure of the vocal folds, just as in other tissues, and that hyaluronic acid (HA) would play a prominent role in these changes. The aim of this study was to investigate the changes in the extracellular matrix of the vocal folds of rats after induction of hypothyroidism.

## Methods

### Ethics

An experimental design with animals was used in this study. The research project was registered and approved by the Ethics Committee for Animals of the Federal University of Sao Paulo under the approval number 2111110422. The study was conducted in accordance with the current regulations of the National Council for the Control of Animal Experiments. The animals were obtained from the Bioterium of the Federal University of Sao Paulo.

### Animals and Research Design

A total of 22 adult female Wistar rats were utilized, which were nonpregnant and nonpuerperal, with no prior medication usage and aged 8 weeks old. The sample size was calculated according to the Experimental Design Wizard of the National Center for Replacement, Refinement & Reduction of Animals in Research (NC3Rs). Data from a previous study on HA concentration in the vocal folds of female rats were used, with a mean difference of 400 ng/mg, a standard deviation (SD) of 320 ng/mg and an effect size (Cohen's *d*) of 1.25.^14^ With an *α* level of 0.05 and a power of 0.80 and assuming an independent samples 2‐sided *t*‐test, a sample size of 12 animals per group was recommended when calculating the sample size. In accordance with the principle of animal reduction, the ethics committee advised reducing the control group. Consequently, the final sample comprised 12 animals in the hypothyroid group and 10 in the control group. All animals were housed in plastic cages (35 cm × 50 cm × 20 cm) at a controlled temperature (21** ±** 2°C). They were exposed to a 12‐hour light‐dark cycle and had free access to water and standard rodent chow.

In the hypothyroid group, hypothyroidism was induced by surgical thyroidectomy. After surgery, the thyroidectomized rats were additionally treated with methimazole (0.03% in tap water) for 8 weeks, and fed a normal diet. The control group (euthyroid animals) underwent a similar surgical procedure, without the removal of the thyroid gland, and received water and a normal diet for 8 weeks. For the surgical procedures, the animals were anesthetized with ketamine (10 mg/kg) and xylazine (100 mg/kg). None of the animals showed stridor or respiratory failure suggestives immobility of the vocal folds or damage to the recurrent laryngeal nerve. At the end of the 8 weeks, the animals were euthanized for sampling.

Following sacrifice, the blood was collected by puncturing the inferior vena cava and centrifuged. The serum was frozen and stored at −70° C for subsequent determination of triiodothyronine (T3), T4, and TSH levels.

The larynx was dissected using microsurgical instruments and a surgical loupe with a magnification of 2.5. After a vertical incision in the cervical skin and the anterior neck muscles dissection, transverse sections made above the hyoid bone and below at the level of the sixth to seventh tracheal ring, with the larynx removed en bloc. The larynx was then isolated from other tissues and opened posteriorly longitudinally along the midline between the posterior cricoarytenoid muscles using microscissors. The vocal folds were dissected taking care to not include the supraglottic and infraglottic areas. The anterior and lateral boundaries were the internal perichondrium of the thyroid cartilage and the posterior boundary were the vocal process of the arytenoid. The sample taken included the entire vocal fold, including the epithelium, LP, and all the muscle.

The vocal folds were frozen in isopentane cooled to a temperature just above freezing and stored at −70°C. One vocal fold from each animal was used for the determination of HA and the other for the measurement of collagen type I collagen, type III collagen, and elastin.

### Hormone Measurements in Serum

The concentration of TSH, T4, and T3 in the serum samples was determined using mouse enzyme‐linked immunosorbent assay (ELISA) kits from Elabscience® (Elabscience Biotechnology Inc.). The manufacturer's instructions were followed.

### Collagen Type I, Collagen Type III, and Elastin HA Quantification

The right vocal folds were placed in 50 µL saline solution (PBS‐Phosphate Buffered Saline) in 1.5 mL conical tubes. The tissue was dissociated using ultrasound (Sonics VibraCell VCX 130 Sonicator) and the tubes were then centrifuged for 8 minutes. The supernatant (protein extract) was collected and stored in a freezer at −80°C. The protein extract was analyzed for the presence of collagen type I, collagen type III, and elastin using the ELISA technique according to the protocol recommended by the manufacturer (BT Lab). Optical density (OD) was determined in a Multiskan SKy spectrophotometer (Thermo Fisher Scientific) using 450 nm filters. The OD values obtained were converted to pg/mL according to the standard curve for each matrix extracellular component. The results obtained were normalized with the total amount of proteins quantified by the Bradford method^15^ and the results were expressed in ng/mg total protein.

### HA Quantification

HA quantification was based on a fluoroassay method and followed the protocol of a previous study.^16^ Standard concentrations (0–500 µg/L) of HA from umbilical cords (Sigma) and aliquots of samples from vocal folds obtained after proteolysis were prepared in a blocking buffer. Subsequently, 100 µL of each solution was added in triplicate to the microwell plates (FluoroNunc maxsorp plates; Perkin‐Elmer Life Sciences) previously coated with a biotin‐conjugated probe and incubated at 4°C for 8 hours. After a wash, the biotin‐conjugated probe was incubated, and after another wash, europium‐labelled streptavidin (Wallac Oy) was added to the system. The streptavidin has an affinity for the biotinylated probe. An enrichment solution (Perkin‐Elmer Life Sciences, Wallac Oy) was added to release the europium bound to the streptavidin and the slides were read in a fluorometer (Victor 2; Wallac Oy). The result for each sample was the average of each triplicate. The HA content was expressed in ng/g dry weight.

### Statistical Analysis

Data were tabulated in Microsoft Office Excel® and measures of centralization (mean and median) and dispersion (standard deviation) were calculated for the corresponding data. For data that did not show a normal distribution, the medians were analyzed using the Wilcoxon Mann–Whitney test. Differences were considered significant if the *P* < .05.

## Results

When comparing between the groups, we found that the hypothyroid group had significantly increased serum TSH concentrations compared to the control group. Serum T4 concentrations were significantly decreased in the hypothyroid group compared to the control group (Table 1). The animals in the hypothyroid group also had reduced T3 values. However, they were not included in the statistics as they were outside the detection range of the test.

**Table 1:** Thyroid Function Profile of Control and Hypothyroid Groups

	Control group			Hypothyroid group			*P* value
	Mean	Median	Sd	Mean	Median	Sd	
TSH (ng/mL)	3.379	3.541	1.714	6.508	7.011	3.841	.0425*
T4 (ng/mL)	61.18	68.48	27.869	18.900	13.210	14.489	.0024*
T3 (ng/mL)	0.515	0.577	0.263	‐	‐	‐	‐

Wilcoxon Mann‐Whitney test.

Abbreviations: mL, milliliter; ng, nanogram; sd, standard deviation.

* *P* < .05.

The concentration of type I collagen was significantly higher in the control group (*P* = .0001) and in the hypothyroidism group (*P* < .0001) than the concentration of type III collagen. When comparing the groups, we found that the concentration of type I and type III collagen was significantly higher in the hypothyroid group compared to the control group. There was no statistically significant difference in elastin concentration between the groups (Table 2).

**Table 2:** Concentrations of Type I Collagen, Type III Collagen, and Elastin (ng/mg) in the Vocal Folds of the Control and Hypothyroid Groups

	Control group			Hypothyroid group			*P* value
	Mean	Median	Sd	Mean	Median	Sd	
Collagen type I	322.66	308.55	87.18	494.13	480.93	197.5	.0432*
Collagen type III	6.71	5.90	2.12	11.59	11.90	4.94	.0339*
Elastin	490.74	476	132.77	594.48	606.25	252.82	.3847

Wilcoxon Mann‐Whitney.

Abbreviations: mg, milligram; ng, nanogram; sd, standard deviation.

* *P* < .05.

**Figure 1:**
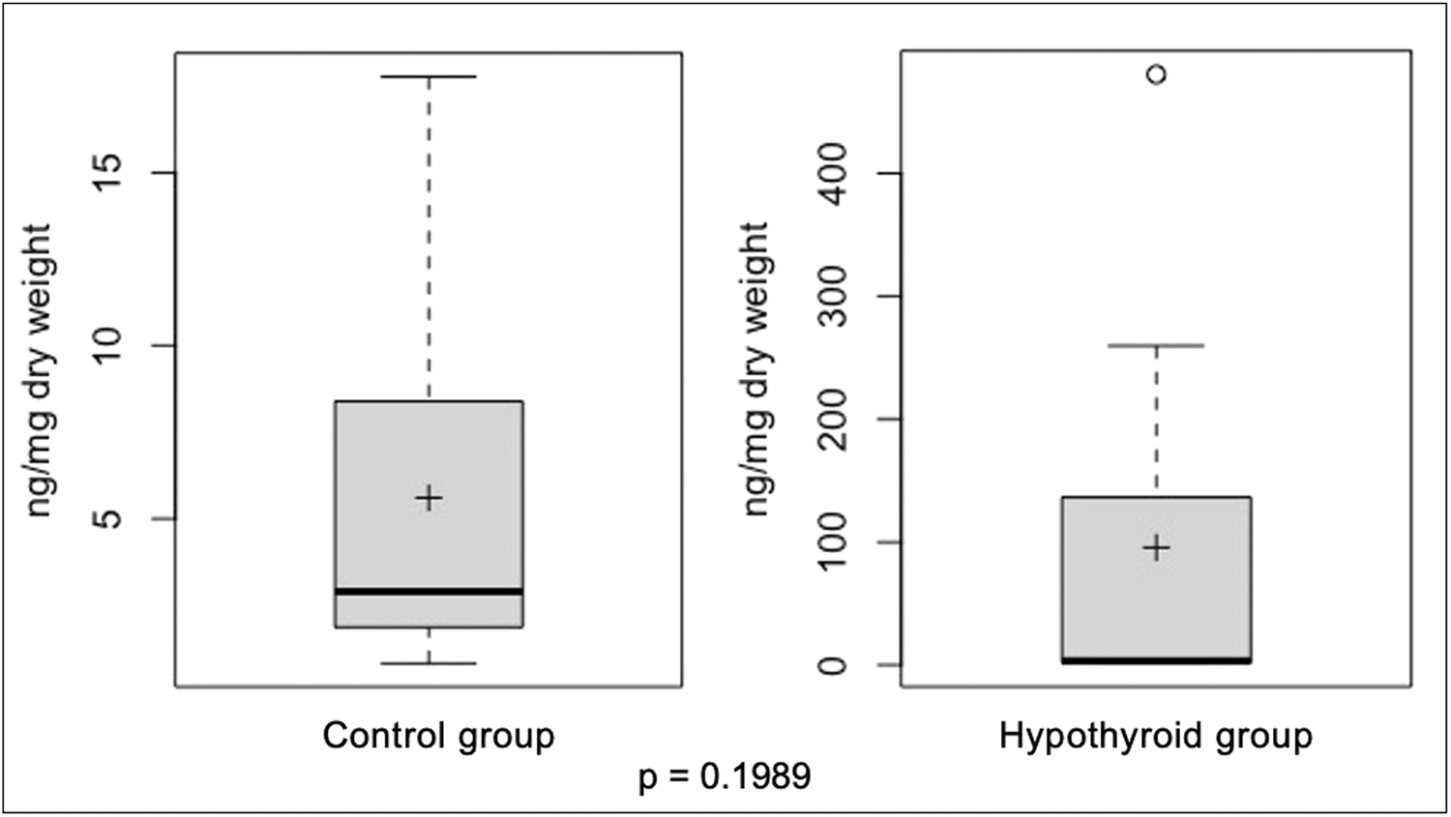
Graphical representation of the concentration of hyaluronic acid in the vocal folds of rats in the control and hypothyroid groups. Wilcoxon Mann‐Whitney test. mg, milligram; ng, nanogram, ‐‐, median; +, mean.

There was no statistically significant difference between the HA concentration in the control and hypothyroid groups (Figure 1).

## Discussion

Our results show that after induction of hypothyroidism, the animals exhibited: (1) an increase in serum TSH concentration, (2) a decrease in serum T4 concentration, (3) an increase in the concentration of type I collagen and type III collagen in the vocal fold, (4) no differences in the concentration of elastin and HA.

We used 2 strategies to induce hypothyroidism in the animals: total thyroidectomy and treatment with 0.03% methimazole. Total thyroidectomy and methimazole treatment complemented each other in inducing hypothyroidism in the animals, and the animals were maintained under these conditions for 2 months. By using a combined model, the occurrence of hypothyroidism is guaranteed, giving you an ideal experimental group. We followed the suggestion of a previous study that proposed a 2‐month postoperative period to investigate the changes in the extracellular matrix of the mature scars of rat vocal folds.^17^ Female rats were chosen because women have a high rate of voice disorders and are the sex with the highest prevalence of benign pathological lesions in the vocal folds.^18^, ^19^

Studies have shown an increase in serum TSH in rats treated with 0.1% and 0.05% methimazole in water for 3 weeks.^20^ We used a concentration of 0.03% to make the water more palatable. Drug treatment guaranteed hypothyroidism in animals that still had residual thyroid tissue after surgery. In thyroidectomized rats and mice, there is a decrease in thyroid hormone levels and an increase in plasma TSH after 20 days of treatment with 0.03% MMI.^21^, ^22^, ^23^ It is known that serum levels of thyroid hormones fall below normal after thyroidectomy and treatment with anti‐thyroid drugs, which increases plasma TSH levels by negative feedback. Our hypothyroid group showed a decrease in the plasma concentration of T3 and T4 and an increase in the plasma concentration of TSH (Table 1).

Collagen is the main component of collagen fibers and a structural protein in the extracellular matrix of various tissues. 28 types have been described, of which type I collagen is the most abundant.^17^, ^24^ Previous studies have shown that type I and type III collagen are the main components of vocal fold LP.^17^, ^25^, ^26^, ^27^ In our study, the concentration of type I collagen was higher than that of type III collagen in euthyroid and hypothyroid animals. In addition, we found higher concentrations of type I and type III collagen in the vocal folds of animals in the hypothyroid group (Table 2). This increase in collagen concentration was also observed in a study in which hypothyroidism was induced in rabbits and in which it was found by histopathologic examination that collagen deposition occurred in the vocal folds of the hypothyroid animals.^28^

The literature shows that the amount of collagen in other tissues increases in hypothyroidism, resulting in greater stiffness or a scarring process that can lead to tissue dysfunction.^6^, ^8^, ^29^ The accumulation of collagen that occurs in hypothyroidism is probably related to a reduction in its degradation.^6^ The extracellular matrix of the surface layer of the LP is directly related to the biomechanical properties that enable vocal fold vibration, and changes in its composition can lead to abnormal vibration and affect voice quality.^30^, ^31^ Considering the importance of collagen in the extracellular matrix of the vocal fold cover and its influence on the vibration of the cover, we hypothesized that an increase in the concentration could be a causal factor for dysphonia.

The present study showed that there was no difference in the concentration of elastin in the vocal folds of the animals in the hypothyroid group (Table 2). This finding is consistent with the literature, which indicates that elastin is only produced to a small extent after puberty and is not easily degraded. Vocal folds disorders involving elastin (benign lesions and scars) are related to its dysfunctional organization or functional decline and not to its production or degradation.^32^ Titze et al.^33^ demonstrated the stability of vocal fold elastin in the face of a vibratory stimulus.

HA is thought to be a causal factor for myxedema, which can occur in the skin and other tissues affected by hypothyroidism.^7^, ^9^, ^34^, ^35^, ^36^ We found no significant differences in the concentration of HA in the vocal folds of the hypothyroid group (Figure 1). However, the presence of an outlier (480.46 ng/mg) caught our attention. This outlier also showed high values in the dosages of collagens and elastin. The analysis of this sample was performed again and the values were confirmed. Fibroblast activity in tissues appears to be subject to complex regulation involving inflammatory mediators and various surface markers.^37^ Thus, in this outlier sample, we can would have another possible factor involved in the etiology of dysphonia that does not occur in all cases. The HA is responsible for maintaining the biomechanical properties of the vocal fold cover and has an influence on the control of fundamental frequency and the absorption of shocks.^38^, ^39^ Although the increase in HA concentration in the hypothyroid group was not statistically significant, the role of this molecule in the development of dysphonia should not be excluded. The HA in the vocal folds appears to be more stable than the collagen. In scarring processes, the HA in the scarred vocal folds remains unchanged for up to 6 months after surgery.^17^ In our study, the time required to activate the maculae flavae and fibroblasts of HA producers and the small number of samples may not have been sufficient, which may have contributed to the failure to increase HA levels.

Some authors consider myxedema, which can occur in hypothyroidism, and Reinke's edema as the same clinical entity.^40^, ^41^, ^42^, ^43^ However, most studies investigating the association between Reinke's edema and hypothyroidism have not confirmed it.^44^, ^45^, ^46^ The different etiopathogenesis and the presence of other changes in the extracellular matrix in hypothyroidism allow us to characterize them as different conditions.

There is little literature about the possible changes in the extracellular matrix of the vocal folds in hypothyroidism. Our study is the first to quantify the concentration of non‐cellular components in the vocal folds of animals with hypothyroidism and compare it with that of normal animals. However, it has its limitations, such as the small number of animals and the fact that the components studied were not quantified by layer, with the results referring to the entire vocal fold, due to the scarcity and size of rat vocal folds. In addition, a longer exposure time seems to be required for the HA concentration to change, but this conflicts with the comfort of the animals. Our results and the findings of previous studies suggest that hypothyroidism leads to a pathological situation of the vocal folds through changes in the composition of the extracellular matrix. Future studies considering structural aspects could confirm this hypothesis.

## Conclusion

Based on our results and limitations, we can conclude that exposure to chronic hypothyroidism cause changes in the extracellular matrix of the vocal folds of adult female rats. We found that the concentration of type I collagen and type III collagen was increased in the hypothyroid animals compared to the control group animals and that there was no difference in the concentration of elastin and HA between the hypothyroid and control groups.

## Author Contributions

**Welber C. Mororó**, conception and design, acquisition of data, analysis and interpretation of data, drafting, final approval, accountable for all aspects; **Karine V. G. de Oliveira**, acquisition of data, analysis and interpretation of data, final approval, accountable for all aspects; **Jônatas B. do Amaral**, acquisition of data, analysis and interpretation of data, final approval, accountable for all aspects; **Gisele Giannocco**, acquisition of data, analysis and interpretation of data, final approval, accountable for all aspects; **João R. M. Martins**, conception and design, acquisition of data, analysis and interpretation of data, final approval, accountable for all aspects; **Noemi G. De Biase**, conception and design, acquisition of data, analysis and interpretation of data, drafting, final approval, accountable for all aspects.

## Disclosures

### Competing interests

None.

### Funding source

This study was financed in part by the Coordenação de Aperfeiçoamento de Pessoal de Nível Superior—Brasil (CAPES)—Finance Code 001.
